# Effect of Fineness of Basaltic Volcanic Ash on Pozzolanic Reactivity, ASR Expansion and Drying Shrinkage of Blended Cement Mortars

**DOI:** 10.3390/ma12162603

**Published:** 2019-08-15

**Authors:** Kaffayatullah Khan, Muhammad Nasir Amin, Muhammad Umair Saleem, Hisham Jahangir Qureshi, Majdi Adel Al-Faiad, Muhammad Ghulam Qadir

**Affiliations:** 1 Department of Civil and Environmental Engineering, College of Engineering, King Faisal University (KFU), Al-Hofuf, Al-Ahsa 31982, Saudi Arabia; 2 Department of Chemical Engineering, College of Engineering, King Faisal University (KFU), Al-Hofuf, Al-Ahsa 31982, Saudi Arabia; 3 Department of Environmental Sciences, COMSATS Institute of Information Technology, Abbottabad 22060, Pakistan

**Keywords:** basaltic volcanic ash, fly ash, pozzolanic reactivity, compressive strength, alkali silica reactivity, drying shrinkage

## Abstract

This study focuses on evaluating the effect of the fineness of basaltic volcanic ash (VA) on the engineering properties of cement pozzolan mixtures. In this study, VA of two different fineness, i.e., VA fine (VF) and VA ultra-fine (VUF) and commercially available fly ash (FA) was used to partially replace cement. Including a control and a hybrid mix (10% each of VUF and FA), eleven mortar mixes were prepared with various percentages of VA and FA (10%, 20% and 30%) to partially replace cement. First, material characterization was performed by using X-ray florescence (XRF), X-ray powder diffraction (XRD), particle size analysis, and a modified Chappelle test. Then, the compressive strength development, alkali silica reactivity (ASR), and drying shrinkage of all mortar mixes were investigated. Finally, XRD analysis on paste samples of all mixes was performed to assess their pozzolanic reactivity at ages of 7 and 91 days. The results showed increased Chappelle reactivity values with an increase in the fineness of the VA. Mortars containing high percentages of VUF (20% and 30%) showed almost equal compressive strength compared to corresponding FA mortars at all ages, however, the hybrid mix (10% VUF + 10% FA) exhibited higher strength than that of the reference mix (100% cement), particularly, at 91 days. At low percentages (10%), ASR expansion in both VF and VUF mortars was higher compared to the corresponding FA mortar and the opposite behavior was observed at high percentages (20% and 30%). Among all the mixes including the control, mortar with VUF was found to be most effective in controlling drying shrinkages at all ages. The rate of consumption of calcium hydroxide (Ca(OH)_2_) for pastes containing VUF and FA was almost the same, while VF showed low Ca(OH)_2_ intensity. These results indicate that an increase in the fineness of VA significantly improvs performance, and therefore, it could be a feasible substitute for commercial admixtures in cement composites.

## 1. Introduction

CO_2_ emissions have increased in the past two decades to such an extent that a sustainable environment for living species on earth has become endangered. Around 25 billion metric tons of concrete is produced every year, which includes 3.3 billion metric tons of Portland cement [1,2]. This high level of cement production has a massive impact on the green life cycle and the environment. According to a conservative estimate, 7% of annual CO_2_ emissions are due to the production of cement clinker, which is the main binding component of concrete [3]. The recent advancements in the cement manufacturing industry and increasing demand for concrete have enabled production units to produce high volumes of cement that pose a significant threat to greenhouse gases [4]. Higher rates of concrete production and consumption have also encouraged worldwide research to study and understand the effect of cement manufacturing on the environment [5].

Given the aforementioned facts, there is no doubt that reducing carbon content in the atmosphere is directly associated with reducing cement content in concrete. Use of supplementary cementitious materials (SCMs) is one of several choices that reduce the use of cement. It not only reduces the use of cement but also increases the durability and design life of the concrete structure [6,7,8]. According to one estimate, to reduce 1 billion tons of CO_2_ emissions per year in the concrete industry, half of the clinkers currently used should be replaced by pozzolans or other low carbon emission cementitious materials [9]. However, the high cost and unavailability of these materials in many parts of world has limited their use in the production of concrete. Therefore, there is a need to further explore naturally available, new materials to fulfill the economic and environmental sustainability demands of the concrete industry. 

Saudi Arabia consumes the highest amount of cement per capita in the world, which demands an annual cement production rate of 55 million tons [10]. Also, the existing infrastructure is prone to weathering and deterioration due to the harsh climatic conditions in the Eastern and Western regions of Saudi Arabia. This deterioration can be reduced by the use of pozzolans, which partially replace the cement content in concrete. In order to meet the demands of the flourishing concrete industry, Saudi Arabia has to import a significant amount of pozzolans and secondary cementitious materials [11]. The western fields of Saudi Arabia contain rich deposits of scoria and basaltic lava [12,13]. To produce environmentally friendly, strong and durable concrete, these locally available materials can be used to replace ordinary Portland cement content. Many researchers have investigated the use of scoria found in Central Harrat Rahat and have validated its suitability for the production of building blocks and lightweight concrete aggregate by using scoria as an additive to ordinary Portland cement [12,14,15]. The term Harrat also encompasses basaltic lava fields that are spread over an area of 180,000 km^2^ in the western region of Saudi Arabia [16]. In the past three decades, these materials have gained popularity due to the significant number of benefits offered by the use of these materials [17,18,19,20,21]. However, regarding the engineering properties of Saudi pozzolanic materials, a sound evaluation of the VA is required to establish their use as partial or complete cement-replacing agents.

Many researchers have investigated the chemical and engineering performance of VA that is naturally available in Saudi Arabia. The chemical and mineralogical properties of VA fulfill the standards set by ASTM. Most of the studies have found that concrete made with up to 30% of VA as a cement substitute has reasonable compressive strength and improved durability properties. They have also found that partial replacement of cement with VA beyond 30% increases the porosity and vascularity in the pore-structure, which causes a reduction in compressive strength and durability properties [22,23,24]. 

The commercially available SCMs such as FA, SF, ground granulated blast furnace slag (GGBFS), and so on are the by-products of different industries and they are obtained through controlled processes. Thus, these products (FA, GGBFS, SF) are highly uniform in nature and having very high fineness and specific surface area (SSA). Such special characteristics made them unique in terms of improving their technical performance. Due to their significant technical advantages, these materials are very popular and highly sought in construction industries. On the other hand, naturally occurring materials such as volcanic ash, volcanic glass, zeolite, etc., present significant variations in their chemical composition and pozzolanic behavior, which is due to the variations in the way they are produced. Previous research indicates that the use of the raw form of these naturally occurring materials is technically not feasible. To cope with this issue, different treatment techniques were recommended such as mechanical grinding to produce the finest material, heating at elevated temperatures to get amorphous phases, and chemical treatment to develop more reactive products [25,26,27,28,29,30,31]. Due to the high cost of these methods, critical research is required to properly investigate all the potential naturally available materials in different parts of the world. To optimize the cost and efficiency, studying the various treatment techniques for specific materials is also required. So far, very limited research is being dedicated to improve the performance of VA by increasing its fineness through severe mechanical grinding. Recently, Khan and Alhozaimy [11] investigated the effect of three different Blaine finenesses (1800, 3400 and 3750 cm^2^/g) of VA on engineering properties of concrete. They witnessed no significant impact of fineness of VA as it did not contribute to improvement of the pozzolanic and engineering properties. However, Al-Chaar et al. [22] studied VA with a Blaine fineness of 3590 cm^2^/g and obtained satisfactory results. Moreover, Celik et al. [23] observed the same mechanical strength at later ages, when VA with the same fineness as that of cement was used in self-compacting concrete. In recent advances, Khan and Amin [32] found better mechanical strength in mortar samples at later ages when ultra-fine VA was used. Patel et al. [33] also observed an enhanced microstructure in paste with an increase in the fineness of VA.

The main focus of the current study was to investigate the effect of two different fineness levels of VA on the engineering properties of a mortar matrix. The two different fineness levels adopted in this study are VA fine (VF) and VA ultra-fine (VUF). To obtain VF and VUF, VA obtained from a commercial supplier was sieved through a # 400 (38 μ) and # 625 (20 μ) sieve, respectively. For the purpose of comparison, commercially available FA was also used in this study as a reference material.

In the first phase of this research, a detailed material characterization of VF, VUF and FA were carried out by performing elemental analysis, phase analysis and particle size distribution analysis. Subsequently, the direct pozzolanic reactivity of all materials (VF, VUF and FA) was determined by using the modified Chappelle test. After the pozzolanic confirmation tests, mortar mixtures were prepared for a control mix (100% cement) as well as for other ten mixes containing VF, VUF and FA as a partial substitute of cement. Six binary mortar mixes containing different percentages (10%, 20% and 30%) of VF and VUF were prepared to study the influence of the fineness. Similarly, three binary mortar mixes containing different percentages of FA (10%, 20% and 30%) were also prepared. In the end, a hybrid mix (ternary) containing 10% VUF and 10% FA was prepared to study their combined effect. For all mixes, test results were compared in terms of their compressive strength development with aging, ASR, drying shrinkage and XRD analysis of their paste samples. 

## 2. Materials and Methods

### 2.1. Materials

**Figure 1:**
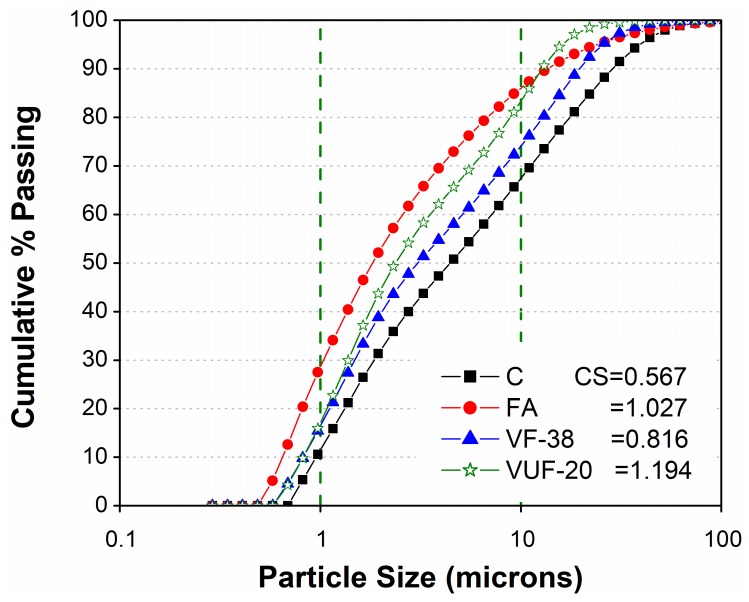
Particle size distribution curves of cement, fly ash, and basaltic volcanic ash of fineness VF (passed through a 38 μ sieve) and VUF (passed through a 20 μ sieve), where, CS represents Microtrac calculated SSA in m^2^/cc [36].

In this study, locally sourced Type-I Portland cement fulfilling the ASTM C150 requirements was used as the main binder [34]. The locally available fine aggregate as per ASTM specification for standard sand with a fineness modulus of 2.54 was used for the preparation of the mortar mixtures [35]. The specific properties of the cement as well as other cement-replacing materials such as VA and FA are given in Table 1. Particle size analysis on samples of cement, FA and VA was performed using laser diffraction Microtrac S3500 with the turbotrac accessory (Microtrac Inc., Montgomeryville, PA 18936, USA, complying with ISO 13320 [36]). The results are shown in Figure 1. The abbreviation CS used in Figure 1 represents the Microtrac calculated SSA of materials in m^2^/cc. A higher CS value means more blain fineness and vice versa. The samples of VA in relation to their different fineness levels after passing through a sieve # 400 (38 μ) and # 625 (20 μ) were identified as VF and VUF, respectively.

#### 2.1.1. Fly Ash (FA)

FA is a byproduct of coal combustion processes that occur in thermal power plants and s currently, it is the most widely used pozzolanic material around the world [37]. In 2006, the estimated production of FA globally was around 500 million tons annually [3]. In general, the incorporation of FA improves the fresh properties of concrete such as workability and compatibility. Besides, it also significantly contributes to the development of the later age strength of concrete and improves concrete durability. It is well-known fact that use of FA up to 25% as a partial substitute of cement produces better later age strength, which is attributed to its improved pozzolanic reactivity at later ages. This is because the high percentage of silica present in the FA reacts with the Ca(OH)_2_ produced by the hydration reaction and forms more calcium silicate hydrates (CSH), which contribute significantly to the later age strength (56 and 91 days). Due to its significant role in enhancing the properties of fresh and hardened concrete, the demand for FA in the concrete industry is always high and hence it is increasing day by day. This is also because the FA, in addition to its structural benefits, contributes indirectly to a reduction in CO_2_ emissions from the cement manufacturing industries, eventually leading towards environmental sustainability.

As mentioned earlier [3], the cement industry is responsible for the 7% of global CO_2_ emissions, which causes global warming and climate change. For this reason, the cement industry has been challenged to minimize CO_2_ emissions. Of all the different technologies, the most effective and straight forward is to substitute cement with SCMs such as FA, SF, GGBFS, and so on. Of these, FA is the most widely used SCM in the construction industry because of the massive quantities available in different parts of the world. In this study, an Indian imported FA that fulfilled the ASTM C618 Type F specifications was used [38].

#### 2.1.2. Basaltic Volcanic Ash (VA)

Twenty-five million years ago, volcanic activity occurred in the western part of Saudi Arabia, which ultimately led to the generation of a massive field of basaltic flows. In the Arabic language, this phenomenon is referred to as “Harrat” [39]. The western part of Saudi Arabia consists of twelve major Harrats and few others that are relatively small, spread along a belt starting from Jeddah, going towards Tabuk through Madinah city. These Harrats occupies a total area of approximately 90,000 km^2^ and consist of several hundreds of scoria cones [40]. Some of these scoria cones have been explored in recent years, and further investigation is still going on to explore many more. Around 490 scoria cones have been explored so far in the areas of Harrat Kishb, Khaybar, Ithnayn, and Kura [41,42]. The Harrat Rahat alone consists of 644 scoria cones [39].

The cones are sometimes entirely made of scoria layers and in some cases consist of alternating scoria layers and lava flows. Scoria materials found along or in the periphery of the cones are lightweight, mostly black, and some reddish color scoria has also been found. The particle size of these materials varies from one place to another and are mostly found in the range between 2 to 32 mm. Previous research studies found that 99% of the scoria materials collected from different cones possess a pozzolanic reactivity as they fulfil the minimum requirements set by different standards for pozzolanic materials [43,44,45,46,47]. Naturally occurring scoria materials, available in vast quantities, have strong potential to be used as an alternative to expensive, commercially available pozzolanic materials in the production of environmentally sustainable and economical concretes.

### 2.2. Mix Proportions and Test Methods

#### 2.2.1. Mix Proportions

In addition to control samples, nine binary mixes were prepared by substituting cement with pozzolanic materials FA, VF and VUF in three different percentages of 10%, 20% and 30%. Besides, a ternary mortar mix, also known as a hybrid mix containing 10% VUF and 10% FA was prepared to examine and investigate the combined effects of VUF and FA pozzolans. According to ASTM C109, the standard water to cement ratio of 0.485 was selected for all mixes, while the sand to cement ratio was kept as 2.75 [48].

#### 2.2.2. Mixing and Testing for Compressive Strength of Mortar Cubes

To prepare uniform mortar mixtures to test for compressive strength on mortar cubes, a three-speed Hobart mixer was used as per ASTM C305 requirements [49]. Cement along with water was first added into the mixing bowl and mixed at a slow speed for 30 s. Afterward, sand was added into the paste mixture and the mixing was maintained by keeping the same speed of mixer for another 30 s. After completing 60 s of mixing, the mixer was completely stopped and its speed mode was changed from slow to intermediate. The mixer was started again and run for 30 s in intermediate speed mode. After a total of 90 s of mixing, the mixer was stopped for 90 s and then started again to continue mixing for 60 s. Immediately after successful completion of mortar mixing, three gang 50 mm^3^ steel molds were filled with fresh mortar in two equal layers as specified by ASTM C109 guidelines. A rubber tamper was used for compacting each layer of the mortar cubes to remove the air bubbles.

**Figure 2:**
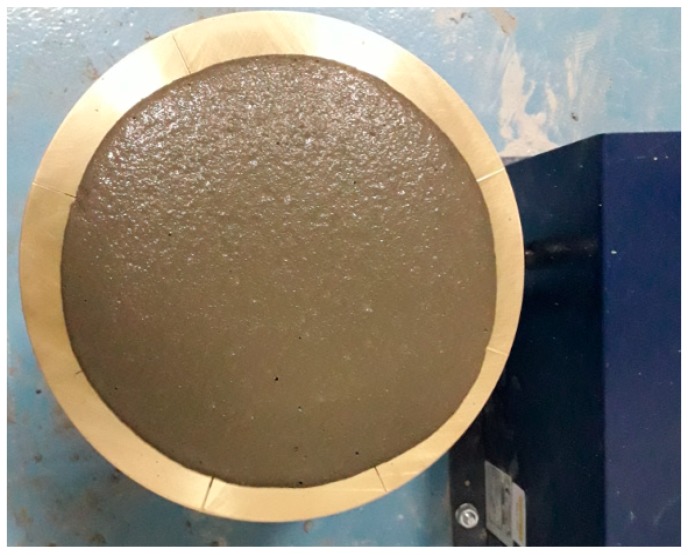
Flow table to measure the flow properties of mixes.

The flow ability of all the mortar mixes was evaluated by using an automatic flow table test according to ASTM C230 and C1437 [50,51]. Figure 2 shows the experimental apparatus used in this study to measure the flow properties of the mortar mixes. Different doses of superplasticizer were used for each mortar mix according to the weight of binder to achieve the minimum flow of 110 ± 5 mm in 25 drops of flow table. The specific values of flow for each mortar mix and its corresponding dose of superplasticizer are given in Table 2. Among the different mixtures, it was observed that both VF and VUF required high doses of superplasticizer as compared to the reference mix (100% cement) to achieve the desired flow. On the other hand, the mixtures containing FA showed better flow ability and, therefore, required the least amount of superplasticizer among all mixes to reach the required flow.

To investigate the compressive strength of each mortar mix, nine identical specimens were cast to test at different ages, that is, 7, 28 and 91 days. Immediately after casting, the fresh mortar cubes were covered with polyethylene sheets to avoid loss of moisture from their surfaces. All the prepared samples were kept for 24 h at a curing temperature of 20 °C. After 24 h of casting, all specimens were demolded for moist curing at 20 °C until the testing age of specimens was reached. 

**Figure 3:**
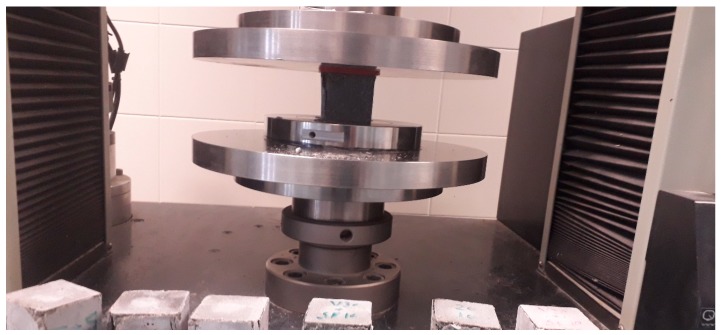
Test set up used to perform compressive strength tests on mortar cubes.

A displacement control QUALITEST QM-300 (Qualitest Inc., Richmond Hill, NC, USA) universal testing machine (UTM), with a loading rate of 1 mm/min was used to conduct compressive strength testing on mortar cubes as per ASTM C109 guidelines. The compression test setup of the UTM used in this study is shown in Figure 3. 

According to ASTM C109 guidelines, the loading rate on each specimen was maintained in a way such that the failure load did not occur in less than 20 s and no later than 80 s. The loading data was recorded automatically through an inbuilt software associated with the UTM. Finally, the ultimate compressive strength was measured for each specimen by dividing the maximum load with the area of the specimen which was 2500 mm^2^. For each mix, three specimens were tested at each specified age (7, 28, and 91 days) to calculate their average values. Thus, the average result of three specimens was noted as the compressive strength of mortar.

### 2.3. Modified Chappelle Test to Evalute Reactivity of Pozzolanic Materials

**Figure 4:**
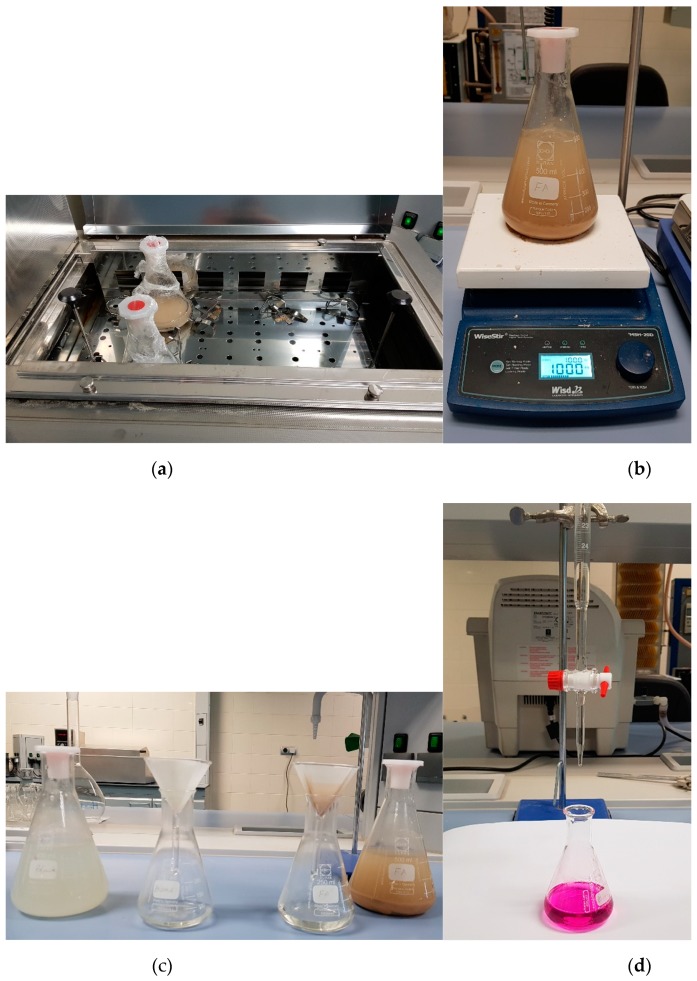
Illustration of the steps performed to determine Chappelle activity (**a**) Samples in the water bath, (**b**) Mixing (sucrose solution), (**c**) Filtration, and (**d**) Titration of the samples.

The Chapelle activity test is a chemical method used to evaluate the reactivity of pozzolanic materials. In this work, the modified Chappelle test was carried out as per NF P 18-531 standards [52]. Figure 4 illustrates the steps performed to carry out Chappelle activity test of pozzolanic materials used in this study. 

As per standard guidelines, 1.0 g of pozzolan material was mixed with 2.0 g of calcium oxide (CaO) in 250 ml of deionized water using a 500 mL Erlenmeyer flask. The flask was sealed and placed in a water bath with continuous shaking at 90 ± 5 °C for 16 ± 2 h using small stainless-steel balls for better mixing. The solution was then cooled down to room temperature and 250 mL of sucrose solution (240 g/L, prepared in deionized water immediately before use) was added to the flasks and was mixed using a magnetic stirrer for 15 min. The solution was then filtered using filter paper and 25 mL of the filtrate was titered with 0.1 M HCl using two drops of phenolphthalein as an indicator. The filtration and pipetting were performed as fast as possible to avoid adsorption of carbon dioxide from the atmosphere. The Chapelle activity was calculated using the following equations:

(1) $$\mathrm{mg}\;\mathrm{CaO}\;\mathrm{per}\;\mathrm{gram}\;\mathrm{of}\;\mathrm{material}=\frac{\left(28\right)\left(2\right)\left(2\right)\left(v_{3}m_{3}-v_{2}\right)}{m_{2}m_{3}m_{4}}$$

where:

*m*_2_: weight of pozzolanic material in grams;
*m*_3_: weight of CaO mixed with pozzolanic material in grams;
*m*_4_: Weight of CaO in the blank sample in grams;
*v*_2_: volume of 0.1 M HCl consumed by the sample solution in milliliters and
*v*_3_: volume of 0.1 M HCl consumed by the blank solution in milliliters.

(2) $$\mathrm{mg}\;\mathrm{Ca}{\left(\mathrm{OH}\right)}_{2}\;\mathrm{fixed}=\frac{\left(2\right)\left(74\right)\left(1000\right)\left(V_{1}-V_{2}\right)}{\left(56\right)V_{1}}$$

where:

*V*_1_: volume of 0.1 M HCl consumed by the blank solution in milliliters and
*V*_2_: volume of 0.1 M HCl consumed by the sample solution in milliliters.

### 2.4. X-Ray Diffraction (XRD) of Pastes Containing Pozzolanic Materials

XRD analysis was conducted on the plain cement paste, pastes consisting of different percentages of VA and FA (10%, 20% and 30%) as well as their blend (20%) by using a Rigaku MiniFlex II X-ray diffractometer (Rigaku, The Woodlands, TX 77381 USA) between 10 and 80°. The step size was kept constant at 0.02 throughout the tests and the Cu Kα radiation level was kept at 30 kV and 30 mA. The purpose of XRD analysis was to evaluate the pozzolanic potential of VA (VF and VUF) and FA samples by comparing the intensities of Ca(OH)_2_ of these materials at different ages (7 and 91 days).

### 2.5. Specimen Preparation and Test Method to Measure Expansion due to Alkali Silica Reaction

**Figure 5:**
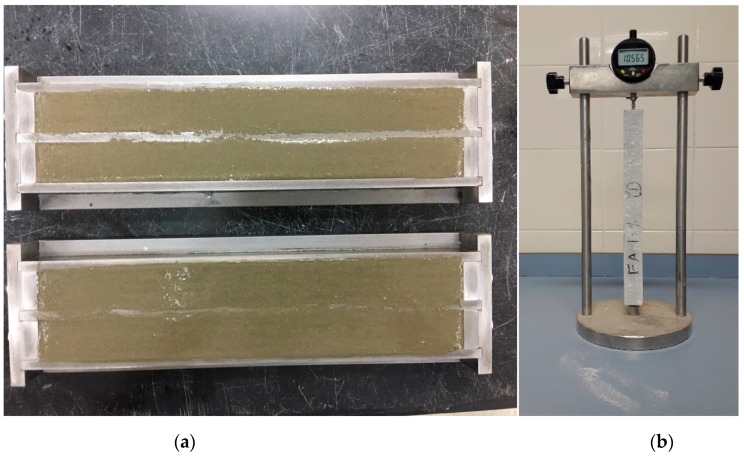
(**a**) Mortar bars after casting for alkali silica reactivity (ASR) expansion and drying shrinkage, and (**b**) test set up to measure length change by comparator for ASR expansion and drying shrinkage.

To evaluate the influence of different pozzolanic materials (VA and FA) on controlling the expansion caused by ASR, expansion tests were performed in accordance with ASTM C1567/ASTM C1260 standards [53,54]. As per the standard, two identical mortar bars (25 mm × 25 mm × 285 mm) with a steel gauge stud at each end were cast for each mortar mix. To activate the alkali silica reaction, the part of natural standard sand used in mixing for ASR was replaced with crushed glass bottle aggregates with sizes ranging from 4.7–1.18 mm [55,56]. The aggregates were graded such that they satisfied the standard requirements of ASTM C1260 for ASR. After the preparation of mortar bars, the molds were stored under standard curing conditions with a temperature of 23 °C and relative humidity of 100% in an environmental chamber. After 24 h of initial curing, mortar bars were removed from the chamber and demolded. All the specimens were labeled according to their mix IDs and initial readings were measured by using a length comparator according to ASTM C490 standard [57]. Figure 5 shows the mortar bars immediately after casting and the length comparator holding the specimen used for reading. After taking the initial readings, samples were submerged in water and kept in an oven at 80 °C for 24 h. Afterward, mortar bars were removed from the oven and measured again considering it as zero reading. Finally, the mortar bars were placed in 1N NaOH solution at 80 °C. The length change of mortar bars was measured up to 16 days and after each reading was taken, they were returned to the same solution. The readings were taken at 3, 6, 9, 12, 14 and 16 days after casting.

### 2.6. Specimen Preparation and Test Method to Measure Drying Shrinkage

After casting the mortar bars for ASR tests, additional mortar bars of the same size (25 mm × 25 mm × 285 mm) were cast to measure the drying shrinkage of the control as well as all other mixes. The drying shrinkage of the mortar bars was measured according to the ASTM C596 standard [58]. For each mixture proportion, a standard mix containing one part of cement and two parts of fine aggregate was prepared to cast two identical mortar bars. Water to cement ratio was kept the same in all mixes to maintain the standard flow as per the standard requirement. After casting, the molds were placed in an environmental chamber under a temperature of 23 °C and 95% relative humidity. Following 24 h of casting and standard curing, mortar bars were demolded and continuously cured in the same environmental chamber up to 7 days. At the age of 7 days, all the mortar bars were removed from the chamber one by one to take their first comparator readings. Subsequently, all the mortar bars were stored under identical laboratory conditions of temperature (23 °C) and relative humidity (50%) until further readings were made at 28 and 91 days. 

## 3. Results and Discussions

### 3.1. Chemical and Physical Characteristic of Basaltic Volcanic Ash

**Figure 6:**
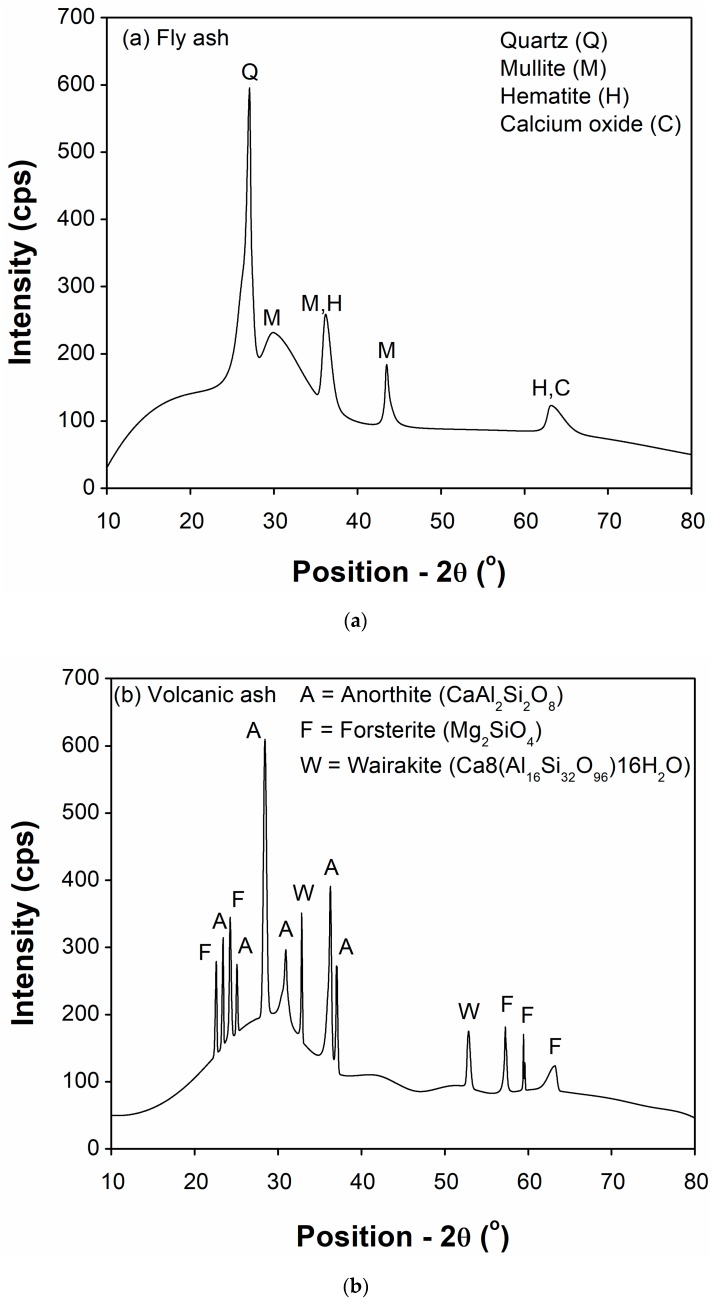
XRD patterns of pozzolanic materials used in this study (**a**) FA, and (**b**) VA.

A comparison of the chemical composition of VA and FA is presented in Table 1, which shows the presence of many oxides; however, the basic primary oxides in both materials are silica (SiO_2_), alumina (Al_2_O_3_), and iron (Fe_2_O_3_) oxides. The sum of these three major oxides (73.6% and 84.7% for VA and FA, respectively) fulfills the minimum requirement of 70% as set by the ASTM C618. Moreover, the XRD diffractograms for both materials (raw VA and FA) are shown in Figure 6.

The XRD analysis of BVA indicates glassy amorphous phases such as anorthitic (CaAl_2_Si_2_O_8_, forsteritic (Mg_2_SiO_4_) and wairakite (Ca_8_(Al_16_Si_32_O_96_) 16H_2_O) as the main phases that are present. On the other hand, XRD peaks of FA shows that it mainly consists of two glassy phases such as mullite (3Al_2_O_3_.2SiO_2_) and quartz (SiO_2_), and two other minor phases such as hematite (Fe_2_O_3_) and calcium oxide (CaO). When used as a cement substitute, the above-mentioned phases of both BVA and FA react partially with cement to form new products, which ultimately contributes to the strength and durability of the cement matrix.

The particle size analysis comparing cement, FA and VA of both fineness (VF and VUF) is shown in Figure 1. It was observed that the particle size of both materials FA and VA (VF and VUF) used in this study were finer than cement. Moreover, the curves indicated VF as less fine than FA and VUF. However, VUF exhibited the highest fineness among all the samples and, therefore, it was expected to act as a filler and contribute towards the pozzolanic reactivity in the cementing system. 

### 3.2. Chappelle Reactivity Test

The Chapelle reactivity test is used as an indicator of how much of the CaO is utilized by the pozzolanas and a higher value means more CaO consumption. As expected, it can be seen from the test results presented in Table 3 that the FA sample demonstrated high reactivity. This must be because the FA is obtained as a result of established industrial processes under controlled conditions. On the other hand, among the VA samples, VUF showed a higher reactivity compared to VF. This indicates that the size distribution of the pozzolan material can have a great influence on its reactivity with CaO. Finally, it can be concluded that the Chappelle test results showed good resemblance with other tests performed in this study for the evaluation of pozzolanic reactivity of different materials (FA and VA) as a partial substitute of cement. In the end, the Chappelle test results were also found to be consistent with the results of other performance tests presented in this study.

### 3.3. Compressive Strength of Control Mortar and Mortars Containing Pozzolanic Materials 

**Figure 7:**
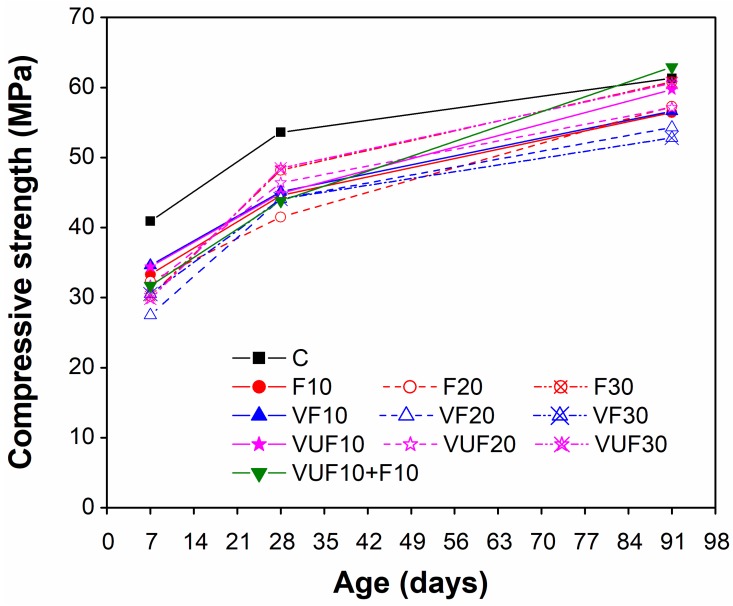
Comparison of compressive strength between control mortar and mortars containing different percentages of pozzolanic materials.

The compressive strength of all mixes was calculated by taking the average of three identical specimens and these are listed in Table 4. The same results were compared in Figure 7 to illustrate the differences between mixes with respect to aging for the different finenesses of VA (VF and VUF) and their different percentages (10%, 20%, and 30%) as a replacement of cement.

From Figure 7, it can be seen that the compressive strength of mortar decreased with an increasing percentage of pozzolanic materials at 7 and 28 days, regardless of their type (VA or FA) or the fineness of the VA (VF and VUF). These results are obviously due to a lesser amount of cement in all mixes containing pozzolanic materials and their low pozzolanic reactivity at early ages. However, the compressive strength of some binary mixes containing 30% FA or 30% VUF increased significantly with aging from 28–91 days due to their strong pozzolanic reaction and reached the compressive strength of the CM at 91 days. Moreover, among all mixes, the only ternary mix containing 10% VUF and 10% FA produced the highest compressive strength at 91 days, even higher than the CM by 3.45%. It is expected that the combined effect of the increased fineness of VUF (˂20 μ) and the high pozzolanic reactivity of FA is responsible for the improved compressive strength of the ternary mix at later ages.

Despite having lower 7 and 28 day compressive strength than CM, all mixes containing FA or VA easily satisfied standard strength activity index requirements for mineral admixtures as specified by ASTM C618. According to ASTM C618, mortars containing cement replacing materials must demonstrate 7 and 28 days compressive strengths more than 75% of CM at the same ages. The strength activity index values were calculated according to ASTM C311 [59].

Although all mortar mixes demonstrated lower 7 and 28 days strength than CM, mortars containing 10% VF or 10% VUF produced slightly higher strength than corresponding 10% FA mortar at all ages. At 91 days, the compressive strength of 10% VUF was 5.48% and 5.85% higher than 10% VF and 10% FA mortar, respectively. This is because of the improved packing effect of finer VA particles in VUF than VF as well as better pozzolanic reactivity of VA than FA at low replacement levels. However, in mortars containing high percentages of cement replacing materials, the compressive strength decreased at 7 days with an increased percentage of VF and VUF (20 and 30%) compared to the FA mortars at same percentage replacements. It kept decreasing with aging in VF (20 and 30%) at 28 and 91 days as compared to FA (20 and 30%) at the same ages, except in VUF where it was comparable to FA mortars having the same percentages (20 and 30%). This indicated that the pozzolanic reactivity of FA was better than VA mortars at high percentages and high fineness of VA is required to achieve strength comparable to FA mortars. A combined effect of both FA and VA on compressive strength development in the form of ternary blends (10% VUF + 10% FA) was also noticeable both at early as well as later ages, particularly at 91 days where it even exceeded the strength of CM. This is attributed to the enhanced filling ability and better pozzolanicity of 10% VUF at all ages as well as the increased later age pozzolanicity of 10% FA. The fine particles of VUF help fill the voids, and thus improve the packing density of the matrix. The XRD results (Figure 6) showed significant amounts of amorphous phases present in FA, while Chappelle tests confirmed the consumption of high amount of Ca(OH)_2_ by FA as compared to VF and VUF. The reaction of amorphous silica and alumina phases with Ca(OH)_2_ would lead to the formation of more CSH, which eventually, contributed to high later age strength development in the ternary mix.

### 3.4. X-Ray Diffraction of Cement Pastes Containing Pozzolanic Materials 

**Figure 8:**
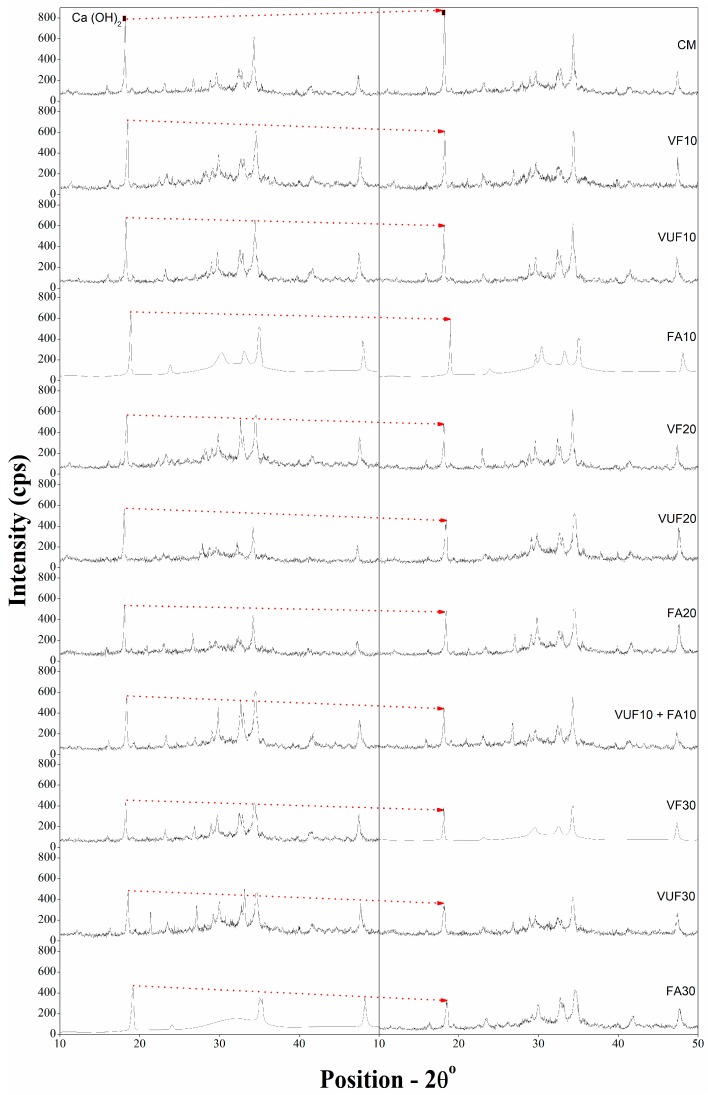
Comparison of XRD peaks between control paste (100% cement) and pastes containing different percentages of FA, VF and VUF at age of 7 and 91 days.

Figure 8 shows the comparison of XRD analysis results for paste samples of control mortar and mortars containing different percentages of both FA and VA (VF and VUF) and their blend (10% VUF + 10% FA). It is well known that the amorphous and glassy silica, which is the major oxide present in the pozzolans, reacts with Ca(OH)_2_ formed by the hydration of calcium silicate (CS). These pozzolans slowly react with the Ca(OH)_2_ present in the cement matrix and form more CSH, which is the main product responsible for strength development. So, the pozzolanic potential of cement substituting materials can be examined by ascertaining the depletion of Ca(OH)_2_ in the cement paste [60].

The XRD pattern of cement pastes of all mixes, as shown in Figure 8 were compared for their peak intensity of Ca(OH)_2_ corresponding to two-theta values of 18.02 degree at 7 and 91 days. The increased intensity of Ca(OH)_2_ in the control paste with aging is obviously due to the continuous hydration of CS, leading to the formation of a high amount of Ca(OH)_2_. However, as compared to control, the intensity of Ca(OH)_2_ at 7 days remained less in all other mixes, irrespective of their type (FA or VA), fineness (VF or VUF) or percentage replacements (10%, 20% or 30%). This, must be due to the lesser amount of cement used in all mixes containing pozzolanic materials. The further reduction in Ca(OH)_2_ peaks with aging from 7 to 91 days demonstrated the pozzolanic reactivity of both FA and VA. This is due to the reaction of their glassy silica with Ca(OH)_2_, which ultimately forms more hydration products [61]. 

More specifically, the XRD results for different pozzolanic materials demonstrated the better pozzolanic performance of both FA and VUF at 91 days as compared to pastes containing VF at all percentages (10%, 20%, and 30%). This means that the rate of depletion of Ca(OH)_2_ from 7 to 91 days in FA and VUF pastes was higher than that of corresponding pastes containing VF. The better pozzolanic performance of both FA- and VUF-containing pastes may be because of their high level of fineness and more SSA, which can lead to increased pozzolanic reactivity and hence the reduction of Ca(OH)_2_. Moreover, further comparison between pastes showed that FA has better pozzolanic reactivity compared to VUF. This was observed because the rate of consumption of Ca(OH)_2_ in the pastes containing FA is relatively higher compared to pastes containing VUF. 

### 3.5. Influence of Pozzolanic Materials on Expansion Caused by Alkali Silica Reaction (ASR) 

**Figure 9:**
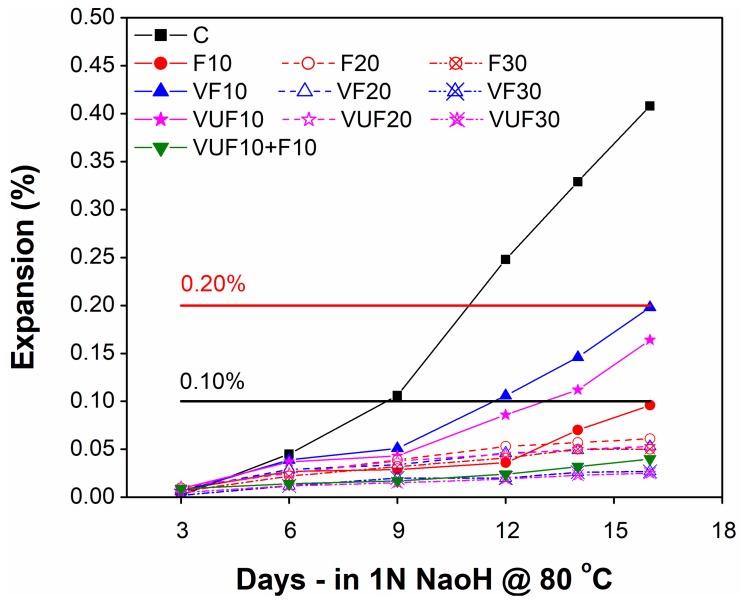
Comparison of ASR expansion between control mortar bar and mortar containing cement, FA, VF and VUF after exposure to accelerated curing conditions.

Figure 9 shows the ASR expansion of accelerated mortar bars for all mixes containing varying percentages of FA and VA with different fineness levels (VF and VUF). According to ASTM C1260, an aggregate is considered to be reactive and will cause potentially deleterious internal expansion provided that the average expansion of three mortar bars at 16 days after casting and curing at 80 °C in 1N NaOH solution is more than 0.20%. The average expansion of the control bar (100% cement) at 16 days after casting was recorded as 0.4%, which indicates the high reactivity of aggregates used in this study.

The comparison of ASR results showed relatively less expansion compared to the control in all mixes containing pozzolanic materials irrespective of their type (FA or VA), fineness (VF or VUF) and percentage replacement (10%, 20% or 30%). Despite relatively less expansion as compared to control, mixtures with low percentages such as 10% VF and 10% VUF still exceeded the expansion limit of 0.1% specified by ASTM C1567 with values of 0.198% and 0.164%, respectively. However, the comparison between 10% VF and 10% VUF demonstrated a significant benefit of greater fineness of VA in terms of mitigating ASR expansion from cement composites. Unlike VA, the mixture containing 10% FA still showed (0.096) better performance in reducing ASR expansion as it produced only 0.096%, which in accordance with ASTM C1567 is within the acceptable limits. Moreover, the result demonstrated a further decrease in ASR expansion with an increasing percentage of cement substitution from 10–20% or 20–30%, either with FA or VA. Interestingly, the mortar mixtures with high percentage replacements of cement in the form of both VF and VUF (20% and 30%) showed better performance in terms of controlling ASR expansion as compared to mixtures containing the corresponding percentages (20% and 30%) of FA. Like 10% cement substitution, the results further demonstrate the continued trend of decreasing ASR expansion with increasing fineness of VA from VF to VUF even at their higher percentages. For instance, the ASR expansion of 20% and 30% VUF mortar bars was lower than the corresponding VF mortar bars. However, the difference in their ASR expansion remained significantly low.

Finally, a hybrid mixture composed of 10% VUF and 10% FA was formulated to obtain the maximum benefit in terms of controlling ASR expansion. It was observed that the ASR expansion was significantly reduced in the hybrid mixture as compared to all other binary mixtures containing 20% pozzolanic material alone (FA, VF or VUF). Overall, the above results suggested that the use of VA as a substitute for cement in low percentages such as 10% may not be effective in controlling ASR expansion. However, cement substitution in high percentages with high fineness VA, such as 20% VUF and 30% VUF can be very effective in inhibiting ASR expansion as compared to both VF and commercially available FA.

### 3.6. Influence of Pozzolanic Materials on Drying Shrinkage with Respect to Aging

**Figure 10:**
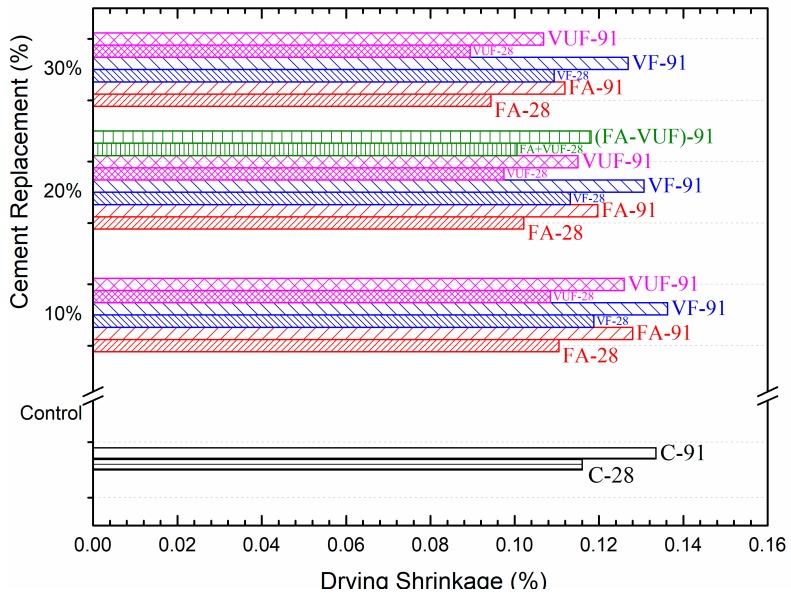
Comparison of drying shrinkage between the control mortar and mortars containing different percentages of FA, VF and VUF at 28 and 91 days.

Figure 10 compares the drying shrinkage of the control mortar and the mortars containing pozzolanic materials. For all the mixes, the mortar bars used to test for drying shrinkage were cured under identical conditions as specified by ASTM C596. The measurement of drying shrinkage was carried out at 28 and 91 days. It can be seen from the results that the drying shrinkage of mortar mixes increased with aging irrespective of the type of pozzolanic material (VA or FA). However, it was found that the drying shrinkage decreases with an increase in the percentage of cement replaced with pozzolanic materials. This must be due to the fact that when the cement was substituted with finer materials, their filling effect reduces total pore volume, and thus causes a reduction in porosity and the drying shrinkage [62].

At 28 days, all mixes showed less drying shrinkage as compared to the control except the mortar containing 10% VF. However, the drying shrinkage in VF mortars also decreased like other mortars containing FA or VUF with an increase in its percentages at 20% and 30% cement substitution. It is worth mentioning that all the mixes containing pozzolanic materials (FA or VS) satisfied ASTM C618 requirements for drying shrinkage. According to the standard, the drying shrinkage strain of mortar made with cement substitute materials should not be more than 0.03% when compared to control samples. The current results showed that the difference in drying shrinkage strain between control and the mixes with pozzolanic materials is well below the ASTM C618 standard limits. More importantly, the mortars with VUF demonstrated even better resistance against drying shrinkage as compared to other mixes such as the control, FA and VF, which showed remarkably less shrinkage at all its percentage replacements with cement irrespective of the aging effects. This behavior can be attributed to its fine particle sizes as it increases the filling ability, improves pore refinement, and thus reduces the drying shrinkage. During the initial stages, it has also been noted by other researchers that the fine particles of the cement substitute materials act as a micro aggregate in the paste and play a vital role in the reduction in drying shrinkage [63,64].

## 4. Conclusions

This work focuses on evaluating the viable use of naturally occurring VA as a partial substitute for cement in the production of an economic and sustainable mortar matrix. Detailed experimental work was performed to examine the pozzolanic reactivity, the development of compressive strength, ASR, and drying shrinkage of mortar containing different percentages (10%, 20%, and 30%) of VF, VUF, and FA. The compressive strength was measured at 7, 28 and 91 days along with XRD analysis on paste samples at 7 and 91 days. The purpose of XRD analysis was to evaluate the pozzolanic reactivity required for validation of strength results. Drying shrinkage of mortar bar samples, cured at standard conditions, was recorded at 28 and 91 days. ASR was measured under accelerated curing conditions by using mortar bar samples up to 16 days from the day of casting. The findings of this study are summarized as follows:

- An increase in the fineness of VA, significantly increased the Chappelle reactivity. On the other hand, FA, prepared under controlled conditions, showed better Chappelle reactivity compared to both VF and VUF.
- The incorporation of VF, VUF, and FA at different percentages (10%, 20% and 30%) resulted in a decrease in the compressive strength of mortar cubes at early ages (7 and 28 days) as compared to control samples; this is attributed to the slow pozzolanic reaction of these materials. On the other hand, compressive strength was significantly improved for all pozzolan-incorporated mixes at a later age when samples were further cured up to 91 days. It was found that at lower percentage replacement (10%), FA showed better compressive strength than both VF and VUF at all ages (7, 28 and 91 days). However, at higher percentage replacement (20 and 30%), mortar containing VUF exhibited almost the same compressive strength as that of FA at later ages (91 days). Moreover, the hybrid mixture (10% VUF + 10% FA) exhibited better compressive strength than all other mixes including control, particularly at later ages (91 days). This can be attributed to the high fineness and increased pozzolanic reactivity of both VUF and FA.
- The intensity of Ca(OH)_2_ as shown by the peak, was reduced in all mixes regardless of type (VA or FA), fineness (VF or VUF) or percentage (10%, 20%, and 30%) of pozzolanic materials. The reduction in the amount of Ca(OH)_2_ is due to the lesser amount of cement used in all mixes, except the control, as well as due to the pozzolanic reactivity of FA and VA. More importantly, the rate of depletion of Ca(OH)_2_ in mixes having VUF or FA was faster compared to mixes with VF at all corresponding percentage replacements. The increased pozzolanic reactivity of both VUF and FA is because of their greater fineness as compared to VF. Similar pozzolanic behavior of VUF suggests that it is a viable substitute for commercially available FA for economical and sustainable concretes.
- The results indicated reduced alkali silica expansion in all mixes containing VA or FA regardless of their percentage substitution with cement, except at the lower percentage (10%) for both VF and VUF, where they did not satisfy ASTM standard limits. Unlike VA, FA was equally effective in mitigating ASR expansion even at lower percentages (10%). As a matter of fact, both VF and VUF showed better resistance against ASR expansion as compared to FA at higher percentages of 20% and 30%. Eventually, the results suggested the utilization of more than 10% VA, irrespective of its fineness, to control ASR related expansion of concrete.
- Just like the findings for ASR expansion, the drying shrinkage of all mortar bars also decreased with an increase in the amount of VF, VUF or FA. The drying strain of all the mixes at 28 days was less than the control sample except for VF. However, all the mixes, including VF demonstrated less shrinkage as compared to the control at 91 days. Moreover, the mortar containing VUF showed the best results against drying shrinkage among all mixes, irrespective of their percentage replacements with cement and aging. This must be attributed to the very fine particle size of VUF, which enhances the filling ability and reduces the pore volume of paste.

## Figures and Tables

**Table 1:** Physical and chemical composition of cement, fly ash and basaltic volcanic ash.

	C	FA	VF	VUF
	Physical properties			
Specific gravity (g/cm^3^)	3.15	2.83	2.64	
Fineness (m^2^/kg) (Blain)	344	−	−	−
Fineness (m^2^/cc) (Microtrac S3500)	0.5670	1.027	0.816 (˂38 μ)	1.194 (˂20 μ)
	Chemical properties (oxides, % by weight)			
SiO_2_	20.9	51.5	46.4	
Al_2_O_3_	5.18	24.3	14.4	
Fe_2_O_3_	3.04	8.87	12.8	
(SiO_2_ + Al_2_O_3_ + Fe_2_O_3_) *	−	84.7	73.6	
CaO	63.9	5.15	8.80	
MgO	1.65	3.50	8.30	
Na_2_O	0.10	2.38	3.80	
K_2_O	0.52	1.47	1.90	
SO_3_	2.61	0.23	0.80	
LOI **	2.51	0.25	2.80	
	Compounds (%)			
C_2_S	52.1	−	−	
C_3_S	19.6	−	−	
C_3_A	8.17	−	−	
C_4_AF	8.81	−	−	

* ASTM C618-15; ** LOI = loss on ignition.

**Table 2:** Flow properties of mortar mixes.

Mix ID	Flow (mm) ASTM C1437	Superplasticizer (% of Binder)
CM	110	1.25
FA10	110	0.95
FA20	111	0.70
FA30	112	0.50
VF10	110	1.60
VF20	109	1.85
VF30	109	2.00
VUF10	109	1.60
VUF20	110	1.88
VUF30	109	2.05
VUF10FA10	111	1.30

**Table 3:** Chappelle test results of FA, VF and VUF according to NF P 18-531.

	Chappelle Activity	
Materials	mg Ca(OH)_2_/g sample	mg CaO/g sample
FA	666.27	911.37
VA ˂ 38 μ (VF)	257.61	853.63
VA ˂ 20 μ (VUF)	336.73	871.51

**Table 4:** Compressive strength of mortars with respect to aging.

Mix ID	Compressive Strength (MPa)		
	7 days	28 days	91 days
CM	40.9	53.6	61.3
FA10	33.3	44.6	56.4
FA20	32.3	41.5	57.3
FA30	30.2	48.2	60.8
VF10	34.6	45.1	56.6
VF20	30.5	44.1	52.8
VF30	27.5	44.1	54.3
VUF10	34.4	44.9	59.7
VUF20	31.7	46.4	57.1
VUF30	29.8	48.5	60.6
VUF10FA10	31.7	43.8	62.9
